# What Are the Implications of COVID-19 on Breastfeeding? A Synthesis of Qualitative Evidence Studies

**DOI:** 10.3390/children10071178

**Published:** 2023-07-07

**Authors:** Evangelia Antoniou, Maria Tzitiridou-Chatzopoulou, Chrysa Voyatzaki, Maria Iliadou, Panagiotis Eskitzis, Maria Dagla, Ermioni Palaska, Eirini Orovou

**Affiliations:** 1 Department of Midwifery, Egaleo Park Campus, University of West Attica, Ag. Spyridonos Str., 12243 Egaleo, Greece; miliad@uniwa.gr (M.I.); mariadagla@uniwa.gr (M.D.); epalaska@uniwa.gr (E.P.); eorovou@uniwa.gr (E.O.); 2 Department of Midwifery, University of Western Macedonia, 50200 Ptolemaida, Greece; mariatzitiridou@gmail.com (M.T.-C.); peskitzis@gmail.com (P.E.); 3 Department of Biomedical Sciences, Egaleo Park Campus, University of West Attica, Ag. Spyridonos Str., 12243 Egaleo, Greece; cvoyiatz@uniwa.gr

**Keywords:** COVID-19, pandemic, breastfeeding, exclusive breastfeeding, breastfeeding support

## Abstract

Introduction: Exclusive breastfeeding until six months of life is the ideal way to feed infants. However, there is a significant number of infants who have never breastfed, despite the beneficial properties of breastfeeding. On the other hand, the coronavirus outbreak had significant effects on people’s health, both mentally and physically, and has also impacted the breastfeeding process. Aim: The aim of this study was to investigate the implication of COVID-19 on breastfeeding through qualitative data from databases. Methods: We searched online databases (PubMed, Google Scholar, PsycINFO) for studies published from 2019 to 2023. ‘Out of the 2598 papers we found, only 12 were included in the review’. More specifically, from the 1558 papers remaining from the title and abstract evaluation as well as duplicates, a further 1546 papers belonging to our exclusion criteria were removed (all types of reviews, letters to editors, and quantitative articles). Results: Our results covered three subjects: breastfeeding support during the pandemic, effects of social containment measures on breastfeeding, and additional outcomes regarding breastfeeding. Most voices found the effects of the pandemic on breastfeeding beneficial, with reduced professional support and a high degree of support from the environment. Additional negative factors were observed, as well as consequences of the pandemic in women’s lives. Conclusions: COVID-19 was the occasion to understand the power of the supportive environment of the woman, especially the partner, in establishing and maintaining breastfeeding. Therefore, policy makers and health professionals, especially midwives, should implement family-centered breastfeeding strategies that are more supportive of the partner role, providing problem counseling when and where deemed necessary.

## 1. Introduction

Exclusive breastfeeding, which entails feeding infants solely with mother’s milk for the first six months, is the optimal way of feeding infants. Then, complementary foods should be introduced into the infant’s diet, along with breastfeeding, until the age of 2 years and above [1]. According to UNICEF, improving breastfeeding rates could save more than 820,000 children under 5 years old worldwide [2]. It is estimated that 21% of infants in high-income countries and 4% of infants in low- and middle-income countries have never been breastfed, given the effects on morbidity and mortality of these infants [2].

On the other hand, the novel coronavirus (SARS-CoV-2) that originated in Wuhan, China in December 2019 spread rapidly throughout the world until February 2020 [3]. On 30 January 2020, the World Health Organization (WHO) officially declared the disease outbreak an international public health emergency [4]. Exposure to SARS-CoV-2 causes pneumonia with increased mortality, while the management of the perinatal period has led to the creation of special protocols [5]. In addition to the economic and social upheavals the pandemic has led to, there have been shocks to the global health system. As a result, hospitals were reorganized into COVID and non-COVID; there were physical barriers to access due to the restriction of movement and fear of infection, which created anxiety of separation from the family [6].

These practices specifically targeted postpartum women infected with SARS-CoV-2 and their infants, with significant knowledge gaps in their management [7]. Infants are more susceptible to COVID-19, exhibiting less severe symptoms than adults; however, they are at higher risk of severe disease and complications [8]. Therefore, special measures had to be taken to limit mother–infant infection. Consequently, immediate treatment in the first hours and care in the following postpartum days significantly affected breastfeeding. Early initiation of breastfeeding allows it to be established more quickly and, consequently, avoid breast milk substitutes. Although breastfeeding has been of particular concern with regard to the transmissibility of the virus to the infant, no studies have confirmed infection through breast milk [9,10,11]. Instead, there are reports of the presence of SARS-CoV-2 immunoglobulin in the breast milk of the exposed mother [11]. 

The WHO recommended that mothers with suspected or confirmed COVID-19 be supported with skin-to-skin contact, exclusive breastfeeding, and rooming with their infants (with hand washing and wearing a mask) [8]. Furthermore, a family member should accompany the parturient in order to provide support during labor and the postpartum period and to promote breastfeeding. Labor should be individualized based on obstetric indications and not be influenced by the presence of COVID-19 unless there are maternal or fetal indications that require immediate intervention. Regarding the postpartum period, the mother and neonate should be discharged 24 h after normal delivery and 2 days after cesarean section [6]. However, the mother–infant separation practices, a result of poor management that mainly targeted SARS-CoV-2-infected mothers, dramatically reduced breastfeeding rates and delayed skin-to-skin contact [12,13]. 

In the last two years, a large part of research concerning COVID-19 and perinatal health has been published. More specifically, rates of severe SARS-CoV-2 disease in infants [14], breastfeeding problems and rates [15,16,17,18], perinatal mental health problems [19], and midwifery care conditions [20] were extensively investigated. We are also able to know that transmission of SARS-CoV-2 through vaginal delivery seems unlikely [21], as well as intrauterine vertical transmission [22] and transmission through breastfeeding [9,10,11]. So far, isolating infants from their infected mothers and prohibiting breastfeeding has not been associated with a reduction in postpartum virus transmission compared to maintaining breastfeeding while taking protective measures [23]. Although there is an abundance of research data on the effects of COVID-19 on breastfeeding, most evidence is limited to quantitative data (surveys and systematic reviews); therefore, a systematic review of qualitative data on women’s breastfeeding experiences during the pandemic seems to be lacking in the literature. 

Understanding how the COVID-19 pandemic has affected the initiation and duration of breastfeeding is crucial to understanding the impact of COVID-19 on perinatal public health but also for the organization of future perinatal care. Based on the above, the aim of this study was to investigate the implication of COVID-19 on breastfeeding through qualitative data from databases. 

## 2. Materials and Methods

This study was performed according to the PRISMA (Preferred Reporting Items for Systematic Reviews and Meta-Analyses) guidelines [24].

### 2.1. Study Population

The population of our study consisted of postpartum mothers who gave birth during the COVID-19 period, whether they were infected or not. 

### 2.2. Exposure/Outcomes

We defined exposure as the postpartum period during the COVID-19 pandemic. We defined outcome as the breastfeeding outcomes (problems that interrupted breastfeeding, exclusive breastfeeding or failure to initiate/maintain) after the social containment measures of COVID-19. 

### 2.3. Inclusion and Exclusion Criteria

The inclusion criteria for the articles were: (a) qualitative article published during the COVID-19 pandemic 2019–2023 that evaluated the relationship between COVID-19 and breastfeeding outcomes; and (b) written in English. However, we excluded papers that (a) were review studies (literature reviews, systematic reviews, and meta-analyses), (b) did not evaluate the relationship between the COVID-19 pandemic and breastfeeding, (c) were written in a language other than English, or (d) had no quality analysis data. 

### 2.4. Study Selection 

We searched all published articles in the following databases: PubMed/Medline, Google Scholar, and PsycINFO, from December 2022 to May 2023. The terms we used were: Search: **(((((((((COVID-19 pandemic)) OR ((SARS COVID 19)) OR (coronovirus)) AND (infant feeding)) OR (breastfeeding)) OR (breastfeeding outcomes)) OR (breast milk)) NOT (reviews)) NOT (quantitative studies))** Filters: **Associated data, from 2019–2023.**

### 2.5. Study Selection

Two authors (E.A. and E.O.) independently screened the databases in the same period, evaluating first the titles and then the abstracts. The full text of the articles was evaluated again using the inclusion and exclusion criteria. They did not need to be reviewed by another author, as there were no disagreements. 

### 2.6. Methodological Quality of the Included Articles 

All studies were assessed for quality using an appropriate quality checklist tool—JBI [25]. The results are shown in Table 1. Out of 12 papers, only 2 received a quality score of 10. The majority of the studies did not include a statement locating the research culturally or vice versa of the researcher to the research. 

## 3. Results

**Figure 1:**
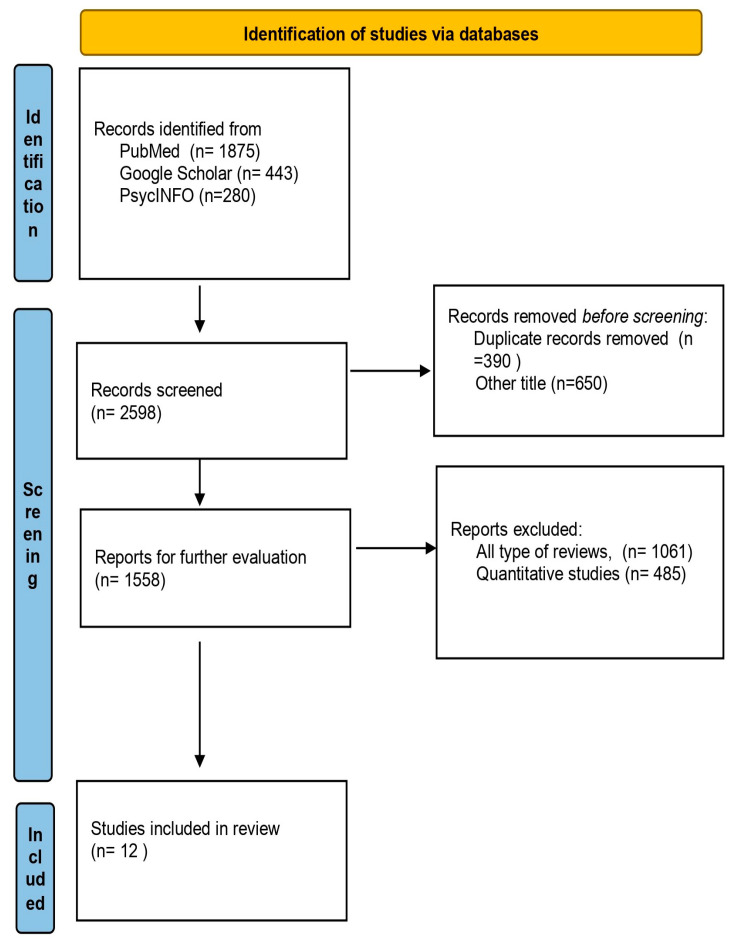
Flow chart of included papers.

The initial search of the databases found 2598 papers. After removing all duplicate and “other title subject” papers, as well as abstract-only papers, 1558 remained to be evaluated further. Then, 1546 papers were removed, as they were reviews, systematic reviews, meta-analyses, letters to editors, or quantitative design surveys. Finally, only 12 papers were included in this systematic qualitative synthesis (Figure 1).

### 3.1. Sample Characteristics 

This study consisted of 12 qualitative studies that presented the impact of the COVID-19 pandemic on breastfeeding. The participants consisted of a total of 2362 mothers, with data collected by semi-structured interviews and two mixed methods. The sample concerned populations from Europe [26,27,28,34,37], Canada [26,36], Africa [34], and Asia [29,30,31,32,33], making it representative of the global population. Interviews were obtained through face-to-face or telephone interviews or via the Internet. 

### 3.2. Theme 1: Breastfeeding Support during the Pandemic

Breastfeeding support was one of the key issues for new mothers in the pandemic. Mothers’ perceptions have common elements between different cultures (Table 2). Mothers generally did not receive support from health professionals for breastfeeding, either during their hospitalization or at home. The issue of understaffing was felt within the hospital. A Norwegian woman reported «……waiting so long for help from the staff» or «during the Breastfeeding support was extremely poor. Obstetric clinics were understaffed» [27]. Regarding the situation during hospitalization, a Spanish woman said «at no time did they tell us anything, we breastfed the child and that’s it” [28].

After discharge from the maternity hospital, one of the main sources of support for women in isolation was primary health care. However, women reported personal social and health care support. Some of the mothers expressed their insecurity about breastfeeding because they did not know if the problems they were experiencing were “normal”. «After we got back … I continued to have trouble breastfeeding. I couldn’t get help from a lactation consultant… because, of course, they didn’t do home visits» [36], a woman from Canada said. « You have to call and make a phone appointment so most of the time I either feel it’s not worth the wait or the hassle to do so. It’s really hard to show people what I’m doing when I’m trying to handle a baby, a breast and a phone», said an English woman [26], while a Spanish woman said, “Everything has been over the phone, and there are things that you need to see in person” [28]. Another Turkish mother said, «They could have called us or shown interest in our breastfeeding … No one called me, do I make myself clear? … They create significant anxiety for a mother. There could have been support that understands the mothers and their emotions» [29].

The very restrictive measures of the pandemic have been particularly frightening for new mothers. This important gap of support from the experts was largely covered by the partner. «Yes, I had my back massaged. My husband discovered the technique on YouTube… My spouse performed the steps. I feel better, and it has the potential to enhance milk supply» [32]. However, mothers received various forms of support upon returning home. The majority of them said that their husbands and families provided them with a lot of support in breastfeeding [37]. «My sister has been my main support; she’s also a mother and has previous experience», said a woman from Spain [28]. But there were also mom networks where mothers found real peer support. A mother from Hong Kong stated, «I am lucky to have joined ‘Hong Kong Moms» [31], «My major supports were the midwife and another mother from the breastfeeding support group» [28]. As it turned out, midwives remained women’s favorite advisors. A woman from Canada said, «I feel my midwives have been very supportive» [26]. However, there were also women who were not supported by the hospital staff; therefore, they described the breastfeeding experience as traumatic [29,36].

### 3.3. Theme 2: Effects of Social Containment Measures on Breastfeeding

In terms of isolation measures, most mothers described the greatest advantage of the pandemic for them was the ample time they had to get to know and bond with their babies and the time to try—despite the problems—to support breastfeeding (Table 2). So, without visitors and unsolicited advice, the women continued to try without worrying about the family’s opinions [26,27,28,30,31,32,33,34,35]. An English woman said, «It was nice to have a little pause before we started seeing people in the extended family because everyone has their opinions……who could say «Does she really feeding so much?» [26]. For some mothers who reported a positive experience, the fear of COVID-19 during the lockdown encouraged them to continue breastfeeding, thereby providing immunity to the infant. «Breastfeeding was easier because they were together all the time» [35] or «I had heard that breast milk has a protective effect from the programs I had watched on TV…» [29].

Breastfeeding is not an easy task and does not always start without problems. Therefore, for some other mothers, isolation did not create the best conditions for breastfeeding [26,36,37]. For example, a mother from Canada said, «the isolation and not being able to have help from your family or friends—I think that was the hardest part» [36] or «… I experienced the first days of my daughter’s life as very traumatic. Due to the COVID-19 restrictions, we also did not receive adequate help and guidance (including breastfeeding support)», from a mother from Norway [27].

In addition to the positive and negative views on the experience of breastfeeding during the pandemic, there were also a few voices saying «nothing has changed» [35].

### 3.4. Theme 3: Additional Outcomes Regarding Breastfeeding

In addition to the aforementioned, there were other positive and negative findings of breastfeeding in the isolation of the pandemic (Table 3). For example, the pandemic increased mother–infant bonding: «I feel very bonded to him and very protective of him» [26]. In addition to the new ways that mothers tried and experimented with breastfeeding without the influence of relatives [30], remote work or less work has played an important role in the establishment and duration of breastfeeding [32,35]. Vaccination against COVID-19 also provided mothers with safety and additional motivation to breastfeed [34], and some breastfeeding support organizations seem to have helped quite a bit [31]. A high educational level in some women worked negatively on breastfeeding (Indonesia) [32], while in other women it worked positively (China) [33].

Some of the negative effects of confinement and problems with breastfeeding appeared in some mothers with long-term effects, such as depressive symptoms [27,36]. A mother from Norway reported, «I’ve been struggling with what I believe is postpartum depression» [27]. Social isolation and concerns about the impact of COVID-19 on their children and themselves were real concerns for Spanish mothers [28], and doubts related to the ambiguous views of health professionals were concerns for Turkish mothers [29].

## 4. Discussion

Discussions among women focused on the effects of the pandemic on breastfeeding. After analyzing the results, it seems that three topics occupied the mothers during the pandemic, such as social support, the effects of isolation, and additional factors that affected breastfeeding during the pandemic. The impact of COVID-19 on the breastfeeding experience was not surprising, given the research that has already been conducted [37,38,39]. A study published in 2020 showed the worsening of breastfeeding problems due to the weaknesses of access to breastfeeding services [40], while in some other settings, COVID-19 policies that implemented separation of the infant from the mother resulted in delayed initiation and cessation of breastfeeding [41].

According to our results, breastfeeding support during the pandemic was carried out mainly by the partner [27,28,30,32,34,35,37]. It is already known that partners can have a strong influence on mothers who choose to breastfeed. More specifically, in a study [42] of fathers who received two-hour training on how to support breastfeeding, mothers were 1.8 times more likely to initiate breastfeeding without problems. In addition, the wider family and friends environment of the mother also has important support in the promotion of breastfeeding [43], and our results confirmed the role of parents and friends of the mother in promoting breastfeeding during the pandemic [28,31,33]. However, researchers from Canada studying the nature of breastfeeding support found that not all types of family support, such as sisterhood, parents, or close friends, are helpful [44]. According to our results, Thai couples were able to experiment with breastfeeding without the influence of older generations on infant feeding [30]. In other words, mothers were given the opportunity to try new styles of mothering without the influence of older members, especially in nuclear families.

However, women considered midwives as important scientific figures, appreciating their emotional support during breastfeeding in the particularly difficult situations of the pandemic [26,28,31]. It is a fact that support from the midwife is linked to the mental well-being of the mother and the exclusivity and duration of breastfeeding [45].

In this systematic review, breastfeeding experiences during the pandemic have been varied. Some women reported that the isolation measures had a positive effect on their breastfeeding experience, allowing them more time with their children. For example, a significant number of women stated that the isolation of the pandemic had a positive effect on the initiation and consolidation of breastfeeding [28,30,31,32,33,34,35]. A possible explanation for this phenomenon is that women devoted themselves fully to the breastfeeding process, working fewer hours at times [32], resulting in an increase in the mother–infant bond [26]. Moreover, breastfeeding as a strengthener of the infant’s immune system, in a significant percentage of women, acted as a motivation to strengthen breastfeeding [29]. However, there were voices that described the impact of the pandemic on the experience of breastfeeding as negative [26,27,29,36,37]. Through these statements, the non-essential treatment of the perceived breastfeeding problems by the health systems, due to the pandemic, is evident. Consequently, the lack of access to breastfeeding support health services forced women to seek the necessary breastfeeding counseling through the Internet and social networks and did not receive the necessary health information [33]. Women feeling that they did not have close contact and face-to-face professional guidance from midwives created a sense of anxiety and hopelessness about their breastfeeding expectations [33].

In addition, in some studies [27,36] there was concern about the occurrence of postpartum depression, as an increase in postpartum depressive symptoms during the pandemic was found. These findings suggest that social isolation, fear of pandemic and disease progression, lack of access to health services, and failure to breastfeed may contribute to adverse psychological outcomes for women in the postpartum period [46].

An important strength of this study is that it is the first to apply a synthesis of qualitative studies to investigate the impact of the COVID-19 pandemic on breastfeeding. The limitations of the study include our lack of knowledge of the mother’s mental health history, the type of delivery, and the quality of the relationship with the partner. In addition, different cultures were investigated, with different health systems and varying educational levels of women, so we do not know how these variables influenced the results.

## 5. Conclusions

The results of our study showed that the COVID-19 pandemic affected mothers in different ways. In the majority of cases, the mothers managed to breastfeed by receiving support from their extended family and mainly from their partner. However, breastfeeding problems, feelings of guilt, and the absence of professional guidance led to the cessation of breastfeeding by many mothers. The results also indicate that COVID-19 was the occasion to understand the power of the supportive environment of the woman, especially the partner, in establishing and maintaining breastfeeding. As many mothers reported that breastfeeding and bonding was easier due to being with their infants without work and visitors, we should consider encouraging all mothers to have quiet time with their infants without distractions. Therefore, policy makers and health professionals, especially midwives, should implement family-centered breastfeeding strategies, providing problem counseling when and where deemed necessary.

## Figures and Tables

**Table 1:** Methodological quality of the included studies.

	Turner [26], 2023, Canada, UK	Vik [27], 2023, Norway	Rodríguez-Gallego [28], 2022, Spain	Aşcı [29], 2022, Turkey	Nuampa [30], 2022, Thailand	Kwan [31], 2022, Hong Kong	Agrina [32], 2022, Indonesia	Yip [33], 2022, China	Igundunasse [34], 2022, South Africa, United Kingdom, Nigeria	Badr [35], 2022, Saudi Arabia	Rice [36], 2021, Canada	Brown [37], 2021, UK
1. Is there congruity between the stated philosophical perspective and the research methodology?	Y	Y	Y	Y	Y	Y	Y	Y	Y	Y	Y	Y
2. Is there congruity between the research methodology and the research question or objectives?	Y	Y	Y	Y	Y	Y	Y	Y	Y	Y	Y	Y
3. Is there congruity between the research methodology and the methods used to collect data?	Y	No	Y	Y	Y	U	Y	Y	Y	Y	Y	Y
4. Is there congruity between the research methodology and the representation and analysis of data?	Y	U	Y	Y	Y	U	Y	Y	Y	Y	Y	Y
5. Is there congruity between the research methodology and the interpretation of results?	Y	Y	Y	Y	Y	U	Y	Y	Y	Y	Y	Y
6. Is there a statement locating the researcher culturally or theoretically?	U	No	No	No	U	U	Y	Y	Y	Y	Y	U
7. Influence of the researcher on the research, and vice versa, is addressed	U	U	Y	U	U	U	U	Y	U	Y	No	U
8. Are participants and their voices adequately represented?	Y	No	Y	Y	Y	No	No	Y	No	Y	Y	Y
9. Is the research ethical according to current criteria or, for recent studies, is there evidence of ethical approval by an appropriate body?	Y	Y	Y	Y	Y	Y	Y	Y	U	Y	U	Y
10. Do the conclusions drawn in the research report flow from the analysis or interpretation of the data?	Y	Y	Y	Y	Y	Y	Y	Y	Y	Y	Y	Y
Overall score	8	5	9	8	8	4	8	10	7	10	8	8

Notes: Reliability questions get the answers: Yes (Y), No, Unclear (U), Not applicable. Yes answers get 1 point.

**Table 2:** Basic characteristics of the included studies.

Author/Year	Participants	Data	Method	Data Analysis	Authors’ Conclusions
Turner [26], 2023 Canada, U.K.	74 women from Canada 24 women from UK	1. Pregnancy–birth cohort 2. Twitter and Instagram accounts	Semi-structured online interviews	Thematic analysis	In both Canada and the UK, new mothers need support from professionals and consistent, reliable health care and social support when breastfeeding.
Vik [27], 2023, Norway	80 women	IMAgiNE EURO	Mixed method via the Internet	Systematic Text Condensation	Compared to pre-pandemic data, there is a decrease in exclusive breastfeeding at discharge during the COVID-19 pandemic in Norway. The findings should alert researchers, policy makers and clinicians in postnatal care services to improve future practices.
Rodríguez-Gallego [28], 2022, Spain	30 mothers	Primary care health centers in Andalusia	Semi-structured interviews by midwives via telephone	Thematic analysis	The use of the Internet to support breastfeeding was an important factor in informing mothers during the pandemic. The role of the midwife was highlighted as quite important. The social restrictions of the pandemic had a positive effect on bundle development and breastfeeding, as a reset of their increased time spent at home.
Aşcı [29], 2022, Turkey	14 mothers	Turkish Ministry of Health	Semi-structured interviews by Health care teams (midwives, nurses and family physicians)	Thematic analysis	Women diagnosed with COVID-19 believed that breast milk would protect their babies and therefore emphasized its continuation. Some mothers refused the treatment due to the fear that it penetrates breast milk. Women would like more support from midwives and psychologists.
Nuampa [30], 2022, Thailand	15 mothers	Self- administered online survey	Semi-structured interviews	Thematic analysis	Informative breastfeeding support from health care providers through the participation of all family members in breastfeeding programs, especially spouses who provide essential emotional support, is a key prerequisite for breastfeeding success.
Kwan [31], 2022, Hong Kong	793 mothers	Online questionnaire	Mixed methods approach	Inductive approach	Giving birth in a public hospital was associated with exclusive breastfeeding. The pandemic has also helped the continuation of breastfeeding with the support of family members and spouses. Paternity leave helped with breastfeeding.

**Table 3:** Data synthesis.

Author/Year	Exposure	Breastfeeding Support	Effects of Social Containment Measures on Breastfeeding	Additional Outcomes
Turner [26], 2023, Canada, U.K.	Breastfeeding during the pandemic and lockdown	By midwives	Negative effect on the initiation and maintenance of breastfeeding	Increased bonding with the infant
Vik [27], 2023, Norway	Breastfeeding during hospitalization, during the pandemic, and lockdown	Lack of professional support Support from partner	Negative effect on the initiation and maintenance of breastfeeding	Fear of postpartum depression was reported by several women
Rodríguez-Gallego [28], 2022, Spain	Breastfeeding during the pandemic and lockdown	Mainly by midwives and family members	Positive effect on the initiation and maintenance of breastfeeding	Concerns about the impact of COVID-19 on their children and themselves, as well as social isolation
Aşcı [29], 2022, Turkey	Breastfeeding during the pandemic and lockdown	From family members and partner	Negative effect on breastfeeding	There was division among health professionals about whether they can breastfeed while on medication. There was general confusion.
Nuampa [30], 2022, Thailand	Breastfeeding during the pandemic and lockdown	From the staff during their hospitalization and from their partner	Positive effect on the initiation and maintenance of breastfeeding	Social isolation for nuclear families provided mothers with the opportunity to try new ways of mothering because of the reduced influence of older generations on infant feeding
Kwan [31], 2022, Hong Kong	Breastfeeding during the pandemic and lockdown	From family members, friends, and midwives	Positive impact on exclusive breastfeeding	Breastfeeding support organizations have been quite helpful. People working in these agencies were allowed to visit the mothers at home.
Agrina [32], 2022, Indonesia	Breastfeeding during the pandemic and lockdown	From family members, mainly by partners	Positive impact on exclusive breastfeeding	The work of the mother played an important role. Women who worked long hours and women with a high level of education breastfed less.
Yip [33], 2022, China	Breastfeeding during the pandemic and lockdown	From friends, traditional practices, online groups	Positive impact on exclusive breastfeeding	Low educational level and low socioeconomic level negatively affected breastfeeding.
Igundunasse [34], South Africa, United Kingdom, Nigeria	Breastfeeding during the pandemic and lockdown	From the partner	Positive impact on exclusive breastfeeding. Mothers wanted in this way to strengthen the health of their children	Most of them had a positive opinion about vaccination
Badr [35], 2022, Saudi Arabia	Breastfeeding during the pandemic and lockdown	From the partner and family	Positive impact on exclusive breastfeeding	Remote working has played an important role in the establishment and duration of breastfeeding
Rice [36], 2021, Canada	Breastfeeding during the pandemic and lockdown	From no one	Negative effect on breastfeeding	Many mothers developed symptoms of postpartum depression
Brown [37], 2021, UK	Breastfeeding during the pandemic and lockdown	From the partner	Negative effect on breastfeeding	Increased bonding with the infant

## Data Availability

Not applicable.
